# Intimate partner violence and drug-addicted women: from explicative models to gender-oriented treatments

**DOI:** 10.3402/ejpt.v5.24496

**Published:** 2014-09-12

**Authors:** Alessandra Simonelli, Caterina E. Pasquali, Francesca De Palo

**Affiliations:** Department of Developmental and Social Psychology, University of Padova, Padova, Italy

**Keywords:** Intimate partner violence, drug addiction, women, treatment

## Abstract

Thanks to studies conducted over the past decades, it has been underlined how harmful consumption of alcohol or other substances and intimate partner violence are intertwined. What has been recognized is, in particular, how the relation between these two factors may be represented as a vicious cycle in which each of them influences the other, reciprocally. The aim of this paper is to offer an overview, firstly, about the global and European scenario of the spread of these constructs, delineating, then, the main explanation models that theorize their connection and those risk factors associated with the environmental settings which may play a significant role. The last part, finally, offers some starting points in order to provide efficient multidisciplinary approaches both to prevent and support victims, increasing their mental, physical, and emotional health.

Over the past decades, it has been highlighted how harmful abuse of alcohol or other substances and intimate partner violence are intertwined. What has been mostly analyzed is how the relation between these factors may be represented as a vicious cycle: indeed, women's consumption of substances may enhance the risk of being abused by their partners, whereas substance use may act as a sort of coping strategy for facing those experiences (Kilkpatrick et al., 1997; World Health Organization [WHO], 2013b).

In particular, what appears as the most relevant characteristic that these phenomena share is the increasing impact they have on mental, physical, and emotional individuals’ health and all those consequences which they may generate inside the social and economic network.

The global picture about the prevalence of drug consumption has shown worrying increasing trends about drug-related deaths and mortality, infectious diseases, and drug treatment demand, indicators that provide important information on the need to adopt strategies and interventions to face these problems. As it has been underlined, indeed, “Illicit drugs continue to jeopardize the health and welfare of people throughout the world. They represent the threat to stability and security of entire regions and to economic and social development. In so many ways, drugs, crime and development are bound to each other” (World Drug Report, United Nations Office on Drugs and Crime [UNODC], 2013a).

The other widespread phenomenon which has concrete and severe consequences on public health, with physical, mental, and reproductive impacts, is the violence against women performed by partners. The latter does not appear as a new scenario, but it is subjected to a growing recognition, because of its role on embracing various aspects of women's life. For this reason, a multidisciplinary approach should be applied, strengthening services and programs for victims and survivors, creating a narrow net of laws, policies and strategies in order to promote awareness and effort (WHO, 2013a).

The report made by WHO (2013a) has highlighted how intimate partner violence, both sexual and physical, is pervasive globally, underlying, also, how many economic and socio-cultural variables play a role in influencing its expression. This paper, specifically, will consider this issue in light of its particular bond with substance addiction, shrinking the focus on a particular sample of women. It has to be remembered, in fact, that not all women who have been subjected to intimate violence by their partners are affected by substance abuse, and, in the same way, that the latter leads always to violence, and vice versa (European Monitoring Centre for Drugs and Drug Addiction [EMCDDA], 2013; UNODC, 2013a; WHO, 2013b).

Firstly, this review offers an overview on the spread of drug addiction and intimate partner violence both in a global point of view and with a narrow focus on the European situation. This work means to delineate, then, how these two ubiquitous phenomena are linked to each other, explaining the three patterns that have been considered as models through which the connection of these constructs takes shape. After this, in the third part, a panorama of the environmental characteristics will be presented of the social context, which have been seen as mediators between the constructs investigated. Finally, in the last part, it will be debated the importance of developing and establishing a treatment approach which involves different services, programs, and roles, in order to be able to provide a useful and efficient support to victims in facing all variables associated to drug addiction and intimate partner violence.

## The extent of the phenomena: a global and European overview

The global picture of the demand for drugs and their use shows that the situation during the past 2 years has been substantially stable, according to UNODC estimates. Furthermore, there has not been any increase in the estimated number of drug users with dependence or drug use disorders, but it is enhancing the total number of users of any kind of illicit substance (World Drug Report, UNODC, 2013b).

In 2011, between 3.6 and 6.9% of the global adult population (people aged between 15 and 64) had used an illicit drug during the previous year, an extent which corresponds to a range between 167 and 315 million people. In the last 4 years, it has been evaluated how the prevalence of the injection of cannabis, opioids, and opiates has substantially increased, whereas the trends of the use of cocaine, amphetamine-type stimulants and “ecstasy-group” substances have declined. What is considered, particularly, as a major public health concern because of its widespread prevalence across all regions is the high level of the misuse or non-medical use of tranquillizers and sedatives, that is even higher than the consumption of illicit substances. Furthermore, a special focus has been highlighted on the increase of the use of new psychoactive substances, considering its extent and the lack of scientific knowledge about their adverse effects (World Drug Report, UNODC, 2013a, see Table 1).

**Table 1:** Estimated number of the extent of drug consumption and drug-related problems among the world general population aged 15–64, 2011

Country	Injecting drug users	HIV among injecting drug users	Drug-related deaths
Africa	997,574	117,502	36,435
America	3,427,561	369,445	52,569
Asia	5,692,005	637,271	104,116
Europe	3,777,948	492,054	15,469
Eastern/Southern Europe	2,907,484	433,836	8,087
Western/Central Europe	870,464	58,217	7,382
Oceania	128,005	1,308	1,957
Global	14,023,092	1,617,580	210,546

Source: United Nations Office on Drugs and Crime, data from the annual report questionnaire, progress reports of the Joint United Nations Programme on HIV/AIDS (UNAIDS).

In the European Drug Report 2013, the EMCDDA offers a wide panorama of the European scenario in which drugs and drug-related harms develop. Differently from the UNODC report, which has investigated the trend and the likely development of the drug use over the past few years, the EMCDDA analysis data has focused on a broader range of time: it shows that at least 85 million adults, around a quarter of the general Europe's adult population, have taken an illicit drug at some point in their lives. At a regional level, it has been reported a high prevalence of injecting drugs consumption in Eastern and South-Eastern Europe (around 1.3% of the population, aged 15–64), where the number of people who use substances is four times higher than the global amount (representing alone the 21% of addicts globally). Particularly, the Russian Federation (2.3%), Estonia (1.5%), the Republic of Moldova (1.2%), Latvia (1.15%), and Belarus (1.11%) are the countries with the highest rates of drug use (EMCDDA, 2013). Furthermore, this report has considered numerous factors which may cause variations in the ways individuals are exposed and may come across illicit substances, such as doses consumed, the co-consumption of other substances, number and length of drug consumption, and individual vulnerability (EMCDDA, 2013).

Even though studying trends of drug addiction considering a gender perspective is a problematic challenge, the EMCDDA in 2006 (EMCDDA, 2006) attempted to focus in more detail on the patterns through which addiction may exhibit itself among women in Europe.

Glancing at the various aspects related to drug use, marked differences appear between the genders: males far outnumber females in all European countries and for the majority of types of drug. Even considering a global view, it has been reported that men are the major consumers of both illicit substances and treatment services.

Despite the fact that the phenomenon is more common among men than women, especially when it is intensive, regular, and problematic, both within a global overlook and within a more circumstantial focus, the gap between men and women is not that vast. Considering cannabis use and binge drinking, a more narrow and equal consumption between the genders appeared in many European regions (EMCDDA, 2006).

Gender differences related to drug abuse are mainly based upon data from patients who are accessing in-treatment programs in Europe: it is hypothesized, though, that the percentage of women drug users who enter services (which is around 20% of patients) is not a realistic representation of the situation (EMCDDA, 2005). This appears to be caused by the various barriers that women, differently from men, may encounter at the first treatment access, considering also that women seem to seek mental health facilities rather than substance abuse treatment programs to face their problems (Lynch, Roth, & Carroll, 2002).

Taking a gender perspective, some patterns of the different phenomenon might be identified in drug use between genders. First of all, the highest number of women among users is found to take tranquillizers, sedatives, and other pharmaceutical drugs (reflecting data from populations in drug treatment), whereas men are more associated with the use of cannabis and cocaine. Another significant pattern has been highlighted studying the different participation in drug use between Northern and Southern European countries: the latter tend to report higher male to female ratios than the other countries in the North. Another relevant pattern is associated with the number of drug-related deaths: far more males than females die due to medical and physical conditions related to a history of drug use. Furthermore, even though the proportions of male and female drug users affecting by hepatitis C are quite similar, HIV prevalence is higher in women (20%) than in men drug users (13%) (EMCDDA, 2006).

Focusing on the extent of the second construct investigated, intimate partner violence, it has been investigated how, worldwide, almost one third (30%) of all women have experienced physical and/or sexual violence by their intimate partner during the course of their relationship and around 38% of all murders of women are committed by their intimate partner (WHO, 2013b). The highest prevalence has been found in African (36.6%), Eastern Mediterranean (37.0%) and South-East Asia Regions (37.7%), whereas prevalence was lower in European and Western Pacific Regions (25.4 and 24.6%, respectively) and in high-income regions (23.2%) (WHO, 2013b).

The report made by WHO also offers a detailed portrait of the effects of intimate violence on women's physical, mental, sexual, and reproductive health: it has been underlined, indeed, that women who have been sexually or physically abused by their partner report higher levels of health problems. For instance, compared with women with no history of intimate violence, they are almost twice as likely to be affected by depression, anxiety or by other mental diseases (for instance, posttraumatic stress disorder [PTSD]); they are also 16% more likely to have a low-birth-weight baby and more than twice as likely to have an abortion; whereas, as mentioned above, they are more likely to contract HIV (around 1.5 times more) in relation to women who have not experienced sexual abuse and violence by their partner (EMCDDA, 2012; WHO, 2013b).

The highest prevalence has been shown in the African regions, where the rate amounts to approximately 37%, whereas the regions of the Americas have reported the second highest prevalence, with around 30% of women who have experienced intimate violence at some point in their lives. Always considering a lifetime exposure, the lower rate has been observed in the high-income region (23%), whereas in the European and Western Pacific Regions, the rate reaches 25% of ever-partnered women.

The European Women's Lobby report (2001) confirms these percentages, underlining that in Europe around one in five women are estimated to experience some form of violence in their lifetime. Moreover, in line with UNODC estimates, almost 70% of women who are victims of homicide each day in Europe are killed by their intimate partner or by another family member (United Nations Office on Drugs and Crime [UNODC], 2013a) and around 3,500 deaths appear to occur in relation to intimate partner violence in 27 of the European Union countries every year, according to research conducted by DAPHNE EU program (DAPHNE, 2007).

Through statistical data from the various reports mentioned here, it is more clearly comprehensible why it is important to contemplate drug addiction and intimate partner violence as phenomena which are always more relevant in our society, especially considering how many factors and consequences may be caused and related to them. Indeed, these two patterns physically and mentally harm the women's health, increasing vulnerability for HIV and other sexually transmitted infections and for various mental diseases, like affective and anxiety disorders, eating behavior disorders, and suicide attempts.

## Theoretical models of substance abuse and intimate partner violence

The association between substance abuse and intimate partner violence has been long studied during the past few decades, aiming to highlight which patterns and most likely aspects may influence their relationship. With this aim, researchers have cast light on three different perspectives, in which substance use and intimate partner violence may be related through a one-way relation, or be reciprocally influenced by each other (Kilkpatrick, Acierno, Resnick, Saunders, & Best, 1997).

In the first model, substance addiction plays the major role, leading addicts to be more vulnerable to assault. According to what is called intoxication-victimization effect (Kaufman Kantor & Straus, 1987), drug and other substances used by victims represent a risk factor which increase the risk of being assaulted: this could certainly occur, firstly, due to the cognitive alterations, such as impaired judgment, memory, misunderstanding of partner's behavior or comments, caused by the pharmacological effects of substances. Secondly, women under the effects of drugs may be more vulnerable because of their impaired ability to recognize and avoid predatory assailants or because of their higher chance to be exposed to the latter, given the environmental setting and lifestyle they share (Kilkpatrick et al., 1997): in support of this, Cottler, Compton, Mager, Spitznagel, and Janca (1992) proved that the odds of the likelihood of being assaulted for substances users were up to 5.06, whereas, similarly, Kessler, Sonnega, Bromet, Hughes, and Nelson (1995) underlined that drug addicts were about 1.5 times in higher risk to experience traumatic events than non-addicts.

In relationships where both partners are addicts, this type of “drug bond” which link the couple may increase conflict between them and, indirectly, abuse. Using, procuring and sharing drugs have, in fact, been underlined as sources of dispute and aggressiveness (Gilbert, El-Bassel, Rajah, Foleno, & Frye, 2001). This kind of setting in which both partners are drug users may also have negative effects on communication skills, facilitating the intensification of violence during arguments (Andrews, Cao, Marsh, & Shin, 2011).

Furthermore, a previous study conducted by Kaufman Kantor and Straus (1987) on a sample consisting of 2,033 female respondents, has also underlined how women are blamed more than men for being drunk or drugged, offering a cause for reflection on gender norms and cultural influences: indeed, cultural approval of violence has been seen as strongly associated with wife abuse, showing, in consequence, how battered women addicts, breaking the norms of appropriate behavior, are viewed as deserving their own beatings. In this study, the percentage report has shown how predictor variables as “wife drunk,” “wife high on drugs,” and “violence norms” were related to, respectively, 46, 24, and 16% of women victims of severe violence. Another aspect suggested by Sandmaier (1980) is the fact that women under effects of substances may become more verbally aggressive, violating another gender norm of appropriateness, which offers a passive and subservient model of female behavior (Kaufman Kantor & Straus, 1989).

The likelihood for women to be exposed to violent intimate behavior has also been seen as influenced by their lack of ability to recognize dangerous signals in interactive exchanges, depriving themselves of effective strategies to face the conflict or moving to safety (Logan, Walker, Cole, & Leukefeld, 2002).

The second pattern that could explain the relation between substance abuse and intimate partner violence considers the first as a consequence of being abused. After an assault, women may look for a strategy to cope with physical, mental, and emotional problems: the negative effects which follow the assault drive individuals to find behaviors that could rapidly reduce negative feelings (Beckham et al., 1995). According to this point conceptualization, Briere (1989) has called this phenomenon *chemical avoidance*: after being subjected to a violence, using or abusing of substances may represent, even if maladaptive and short-lived, a coping strategy through which lowering the amount of physiological and subjective aversive emotions. In light of these theorizations, the observed association between intimate violence and substance abuse would reflect a process of self-medication following the victimization and confirm the tension reduction models of addiction (Conger, 1956; Khantzian, 1997; Testa, Livingston, & Leonard, 2003). Objective support of this model has been provided by Polusny and Follette (1995), who found that a range between 27–37 and 14–31%, respectively for alcohol and drugs use, of assaulted women have lifetime substances-related problems, in contrast with non-abused women (4–20 and 3–12%). Similarly, Burnam et al. (1988) reported that 16% of participants of a probability sample developed substance abuse after a traumatic events. A more recent investigation, conducted by Testa et al. (2003), with 724 women in heterosexual relationships, showed that their use of both marijuana and hard drugs were associated to higher likelihood of experiencing intimate partner violence.

Other theories have explained the connection between substance abuse among women and social relationships: one of those is called *relational model* which posits that substance use is primary used to face the pain and the sufferance that grow up in distorted relationships. The latter, according to this perspective, may be characterized by non-mutuality, isolation and shaming, limiting relational images, abuse, violation or systemic violence, and distortion of sexuality (Convington & Surray, 1997). Seeking ways to maintain and/or establish intimate relations with others, women may depict drug use as a functional, although unhealthy, strategy (Convington, 2002).

Furthermore, women addicts are more likely to develop positive expectations of the effects of substance use, because of their tendency to interpret it as an intimate relationship maintenance mechanism, even if it offers just a temporary relief (Keane, 2004). In support of this perspective, a study conducted by Peters, Khondkaryan, and Sullivan (2012) on a sample of 212 community-based, IPV-exposed women found correlations between substance use expectancies and the severity of physical and sexual IPV, pointing, also, the attention on the association alcohol and drugs expectancies and psychological distress (e.g., PTSD and depression).

Both of these models suggest that the connection between the constructs follow only a unidirectional path, forgetting to consider the likelihood that a reciprocal association may be involved: in order to overshoot the limits of these conceptualizations, the last and more recent perspective focuses the attention on how the linkage between intimate violence and drug addiction among women may be seen as a vicious cycle in which they have both an effect on each other reciprocally.

Violent or sexual assault, in fact, enhances the risk to engage in addictive behavior (Ireland & Widom, 1994), whereas, in turn, the latter intensifies likelihood of being re-victimized by partners (Burnam et al., 1988), with the consequence of increasing the substances seeking by women. This bidirectional relation between substance use and violence shows how both factors are involved in a “cycle of spiraling losses and increasing vulnerabilities” (El-Bassel, Gilbert, Wu, Go, & Hill, 2005). In this vicious circle, re-victimization plays the significant role which links past experiences of substance-related violence with the risk of further additional abuse (Andrews et al., 2011; Rivaux et al., 2008).

Kilkpatrick et al. (1997), attempting to provide evidence on a clear directionality of the relationship between substance abuse and IPV, conducted a three-wave longitudinal study, involving 3,006 women who were followed for 2 years. What this research reported is that drug abuse at Wave 1 is more likely to increase odds of being assaulted in the following 2 years; reciprocally, at Wave 3, odds of substances abuse were critically increased among both women who had or had not been assaulted by partners.

## Risk factors of IPV and substance abuse association

Although several studies have revealed a connection between women's substance addiction and the experience of intimate sexual and/or physical violence from partner, the temporal sequence of these factors has not been clearly determined.

Even though it has been trying to provide evidences on how drug use leads to experiencing violent assault by a partner, it is not women's addictive behavior itself that enhances the vulnerability to be victimized. Instead, it seems that the association between these variables may reflect the mediation of a third factor: antisocial behaviors, difficulty handling conflict or life stressors, risky lifestyle, irritability, and impaired social relational abilities, that are commonly associated with both drug addicts and violent relationships, may significantly influence the impact of the variables on each other (Testa et al., 2003). In line with what was suggested by Kilkpatrick et al. (1997), the potential exposure to violence on women addicts is mainly associated with a risky lifestyle and the environmental setting in which drug users are involved, creating a sort of illegal and socially deviant subculture where violent men are included.

James, Johnson, and Raghavan (2004) have examined in detail the role that environmental aspects may have on the development and the establishment of violent behaviors and drug-related problems. Contextualizing violence and drug abuse may become a crucial factor to structure and foster functional and efficient treatment programs. Thanks to this study among poor populations and neighborhood networks, these authors have focused their attention on those structural conditions, as living in poor neighborhoods and having limited working opportunities, which may shape and influence the relationships and interactions between people at a community level (Cattel, 2001). Women who are strictly wrapped in a limited social world, “intimately tied to and powerfully shaped by their neighborhoods” (James et al., 2004), are surrounded by economic, emotional, and psychological aspects, which heavily influence their lifestyle. Thus, an impoverished neighborhood may become a complex system where the roots of substance abuse and drug-related violence are embedded: in this risky scenario, women are constantly exposed to drugs, increasing the likelihood to being initiated to them by their partners and, consequently, enhancing the risk of being assaulted in the domestic context. Due to the limited number of resources and social support which the networks may offer to them, women appear as socially isolated and often hemmed-in life-threatening situations.

Attempting to explain the effects of social conditions, James et al. (2003) coined the *Structural Violence Para*digm, in which violence is seen as “a set of relations, processes, and social conditions that embody and produce violence and encompasses the conscious and nonconscious view, attitudes, and actions that create everyday social realities.” Violence is represented as any kind of barrier that may limit or prohibit a person from reaching positive living conditions. According with this point of view, the environmental layout (e.g., impoverished neighborhoods), the material arrangements (e.g., no economic or social support resources), through the influence of a cultural lens (e.g., stereotypical representations of social groups), may act as a major role in those processes, which have a relevant impact on the connection between drug use and violence. This theorization is capable of considering these patterns together with all the range of shades which builds their interconnection, from an individual to a societal level, through a relationship and community perspective—levels that WHO has underlined as to be deeply connected to intimate partner violence risks factors (WHO & LSHTM, 2010, see Table 2).

**Table 2:** Risk factors related to intimate partner violence

Perpetration by men	Victimization of women
Individual level	
Demographics	Demographics
Young age Low socio-economic status/income Law education Unemployment	Young, age Low socio-economic status/income Low education Separated/divorced marital status Pregnancy
Exposure to child maltreatment	Exposure to child maltreatment
Intra-parental violence Sexual abuse Physical abuse	Intra-parental violence Sexual abuse
Mental disorder	Mental disorder
Antisocial personality	Depression
Substance use	Substance use
Harmful use of alcohol Illicit drug use Acceptance of violence Past history of being abusive	Harmful use of alcohol Illicit drug use Acceptance of violence Exposure to prior abuse/victimization
Relationship level	
Educational disparity Multiple partners/infidelity	Educational disparity Number of children
Relationship duality	Relationship quality
Marital dissatisfaction/discord Gender role disputes Marital duration	Marital dissatisfaction/discord
Community level	
Acceptance of traditional gender roles	Acceptance of traditional gender roles
Neighborhood characteristics	Neighborhood characteristics
High proportion of poverty High proportion of unemployment High proportion of male literacy Acceptance of violence High proportion of households that use corporal punishment Weak community sanctions	High proportion of poverty High proportion of unemployment High proportion of female literacy Acceptance of violence Law proportion of women with high level of autonomy Law proportion of women with higher education Weak community sanctions
Societal level	
Traditional gender norms and social norms supportive of violence	Divorce regulations by government Lack of legislation on intimate partner violence within marriage Protective marriage law Traditional gender norms and social norms supportive of violence

Source: World Health Organization and London School of Hygiene and Tropical Medicine (LSHTM) (2010).

## Gender-related approaches for treatment

Data from literature and international clinical practice have identified how the trends of the access to the treatment by women and men show significant differences. It has been underlined, in particular, how there are several barriers that women have to face when they seek help and support. The first critical step in these processes is to provide them with a unique array of services which may increase their safety and their personal and social abilities: indeed, as a consequence of their history of abuse, women who have experienced intimate partner violence have specific and more complex needs. After being embedded in dangerous relationships, they may not initially benefit from standard substance abuse treatment programs or from other focused interventions, because of their urgent research of feeling safe (Andrews et al., 2011).

Women who have been psychologically, mentally, and physically alienated, intimidated and humiliated by partners, need, principally, to be welcomed and accepted in the services settings, which are often calibrated on male users characteristics. An unattractive environment has, in fact, been highlighted as one of the most influential aspects which determines why female patients access these services less than males in treatment (Brentari et al., 2011). According to this, talking about treatment perspective, services should provide well-designed spaces and specific attention to women's needs, given also those barriers and gaps which have been identified as absent. In particular, the lack of childcare facilities and child protection issues, weak maternity interventions and attention on ethnic minority women, poor social support network, scarcity of women-only services and the professionals’ negative attitudes toward patients (Becker & Duffy, 2002). Several analyses on the effectiveness of women's treatment programs for drug abuse, indeed, have confirmed how better outcomes are related with attending women-only services (Ashley, Marsden, & Brady, 2003; Orin, Francisco, & Bernichon, 2001).

In light of this, focus should be placed on the development and the establishment of operational residential treatment and social reintegration processes, in order to integrate, simultaneously, intervention on substance abuse, personal characteristics, and community and environmental patterns. Thus, a series of strategies could be created, providing a friendly context in which marginalized women may seek their own psychological, mental, and physical safety.

**Fig. 1:**
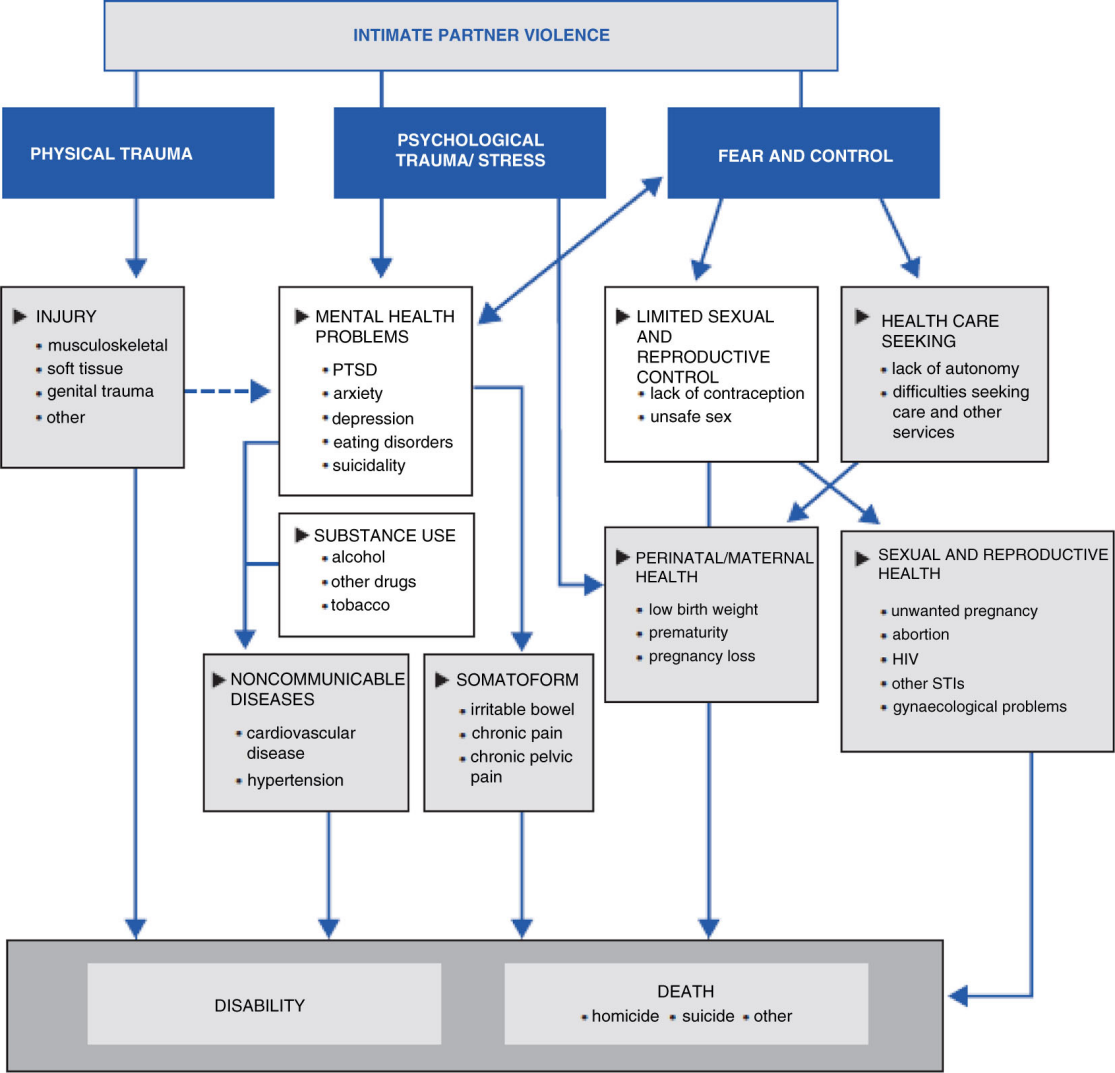
Pathways and health effects on intimate partner violence. Source: World Health Organization, London School of Hygiene and Tropical Medicine (LSHTM), and the South African Medical Research Council (2013).

A multi-agency approach is especially important to establish an effective program: laying the foundations for a collaboration between police, other officers of the justice sector and health and social services may prevent women's negative behaviors and attitudes, intervening early on both violent episodes and drug consumption (Haider, 2008). From the graphic below, the interconnections between these patterns might be various and they may lead to different adverse health outcomes, following risky pathways and mechanisms, from physical and reproductive aspects, to mental and psychological problems (Fig. 1).

Interventions, referenced to rehabilitation programs, should also be addressed to the post-treatment phase; issues particularly significant, indeed, involved the development of employment skills which could help women to reintegrate themselves in the economic net, simultaneously providing educational background and abilities with transversal social competences. Furthermore, offering secure stable transitional housing could also help them to recreate a more positive context to rebuild a new lifestyle, especially if their children are involved (EMCDDA, 2006).

An aspect significantly relevant, in fact, consists of all those patterns which are related to pregnancy and maternity: women addicts who have experienced intimate partners’ violence need to be sustained throughout these processes, offering them a coordinated and integrated healthcare assistance, with substance misuse treatments, harm reduction counselling as long as specific information about both antenatal and postnatal care. Mother–child programs and/or relationship activities, the development of a child-friendly environment, unite to psychological and social support for both mother and child could significantly create a more safe and healthy setting where a family could live. Pregnancy and motherhood, for many women, could represent strong motivators to enter the treatment and to stop the use of drugs, emphasizing the relevance of this aspect for building new treatment opportunities for women (Brentari et al., 2011).

Another aspect is the need to empower women and encourage them to adopt safer behaviors, in order to be capable of facing and recognizing partners’ harmful attitudes. In particular, female drug addicts, if they are forced to perform sexual behaviors or if they trade sex for getting drugs and money back, are more likely to engage in unprotected sex, even with multiple risky sexual partners, and therefore at a higher risk of becoming infected with HIV and other sexually transmitted infections. In light of this, a gender-based approach should focus on the importance of structured STI prevention and treatment programs, to provide more positive coping strategies to face the risk for infections (Brentari et al., 2011).

Given the high levels of psychological and physical issues in women who suffer from substance abuse addiction, a dual diagnosis should be evaluated for a comorbid disorder, in order to diagnose a proper mental health and drug abuse intervention. The main goals of a treatment for dual diagnosis should primarily involve the reduction of psychiatric symptoms and substance use and, simultaneously, be able to improve women's quality of life, establishing stable and appropriate care, with special attention on working and relational settings. Various treatment models may accomplish these goals: in particular, a biopsychosocial approach, which includes motivational interviewing, cognitive behavioral therapy, and social skills training, may represent a valid path to target relevant issues such as motivation, cognition and social skills. In addition, assertive community, case management interventions, and psychosocial treatment may equip women of external support (Brentari et al., 2011). Some studies have investigated the effectiveness of integrated treatment approaches for both substance abuse and intimate partner violence (Fals-Stewart et al., 2002; Fals-Stewart and Sherrod, 2009; Kraanen et al., 2013): they assume that if intoxication from substances and relational dissatisfaction are precursors to the occurrence of experiencing intimate violence, programs should primarily focus on this issue, reducing first of all aggression between partners. An example is provided by the Behavioral Couples Therapy (BCT) (O'Farrel & Fals-Stewart, 2000), a protocol designed for reducing substance use and relationship problems and which has been observed to decrease the proportion of dyads in which men perpetrate violence against their partners in the first year after treatment, showing higher effectiveness in respect to the Individual-Based Therapy (IBT) (Fals-Stewart et al., 2002). Nevertheless, researchers have suggested to include in this approach as some form of individual or group-based therapy, in order to integrate and implement patients’ history (Fals-Stewart & Kennedy, 2005). A pilot study has also been conducted in The Netherlands, which compared the effectiveness of a combined treatment for both substance abuse and intimate violence cognitive behavioral group therapy (CBT) (I-StoP) to a 12-step facilitation group that was not addressed to violence. Results showed that both of them were effective in lowering substance addiction and in reducing male aggressive behaviors, with no relevant differences, suggesting that the second approach could represent a more valid and applicable protocol, given the limited amount of additional therapist's training and the possibility to carry it out at the same institution (Kraanen et al., 2013).

Moreover, several studies have been conducted in order to find evidence about how a gender-based approach in prevention interventions may foster the establishment of women's positive lifestyle (Schinke, 1994). Despite studies on the effectiveness of prevention programs that have demonstrated that only one strategy can be classified as “effective” in preventing violence against women that is using school-based program to prevent dating violence, there are several other protocols which may play a role in decreasing aggressive behaviors and reducing sexual violence either (WHO & LSHTM, 2010). Especially, it has been investigated how intimate partner violence might also be reduced through the activation of primary prevention programs addressed at decreasing the range of all potential harms caused by substances (Anderson, Chisholm, & Fuhr, 2009). Primary prevention strategies should be programmed in line with the main life stages: it is the effectiveness of programs that may be influenced by people's developmental and growth features. In childhood and early adolescence, for instance, home-visitation and parent-education programs have been identified as potential buffers of intimate violence capable of preventing child maltreatment enriching parental functioning skills (Foshee, Reyes, & Wyckoff, 2009), whereas, in adulthood, the decrease of the likelihood of perpetrating violence is mainly owed to the treatment for emotional and conduct behaviors (Meltzer, Gatward, Corbin, Goodman, & Ford, 2003). Moreover, dating violence prevention programs, targeted at the establishment of intimate relationships have been observed to be able to reduce violence, both physically and sexually in adolescence and early adulthood (Foshee et al., 2008), acting also on the occurrence of other health-compromising behaviors, such as substance abuse (Wolfe et al., 2009). Significantly, in adulthood, empowerment and comprehensive programs highlight the role of both individuals and communities as agents of social change in establishing a supportive climate for positively evolving attitudes and behaviors. The most important characteristic of this approach is its multifaceted combination of education and training, participatory rapid needs assessment, public awareness campaigns, and community action (Lankester, 1992). Given the mutual relationship between intimate violence and substance abuse among women, acting on their psychosocial surrounding patterns may lead to build polyhedric approaches and to shape reforms and policies better integrated, strengthening a tendency to focus on changing cultural norms, behaviors, and attitudes as well.

It has been investigated that for effective prevention, women, in contrast to men, prefer to participate in workshops, thanks to the informal type of exchange and extroverted forms of expression which are involved (EMCDDA, 2006), whereas family supervision has been highlighted as a protective factor to avoid substance abuse among girls (Sale, Sambrano, Springer, & Turner, 2005). An example of a gender-specific intervention which has been put in practice in some European countries (Austria, Germany, and the Nordic Countries) has involved workshops and seminars in which boys and girls were split, trying to encourage identity and positive body image development, self-reliance, and action competence (EMCDDA, 2006).

An interesting and innovative study involving seven community-based outpatient treatment programs in various places across the United States has explored the effectiveness of two different interventions directed at women who both experienced intimate partner violence and who suffered from substance use disorders and PTSD (Cohen, Field, Campbell, & Hien, 2013). Participants (*N =*288) of this research were randomly assigned to a *Seeking Safety* Treatment (Najavits, 2002), a short-term therapy, using cognitive behavioral strategies capable of reducing both substance abuse and the negative effects of trauma, and to the Women's Health Education (Miller, Pagan, & Tross, 1998), a psychoeducational intervention which provides theories and techniques about issues particularly pertinent to women's health (e.g., pregnancy, childbirth, sexual behavior, female anatomy, and nutrition). Even though results showed that *Seeking Safet*y is more effective on decreasing risk of future aggressions and impacting on outcomes related to PTSD and substance use, both interventions are important because of their attention on the linkage between different patterns.

Considering the Italian context, in particular, the Municipality of Venice has established the Anti-Violence Network, a permanent group of professionals from various agencies and organizations linked together by the goal of promoting a set of integrated provisions that may answer women's needs. Since 2004, this project has contributed to focusing on planning strategies and services specifically addressed to violence against women and children (Brentari et al., 2011). Furthermore, in the Veneto Region, intervention measures for female drug addiction has been rethought, modifying the functioning of therapeutic communities. In particular, these provide residential care and give hospitality to drug addict women to which a comprehensive rehabilitation pathway is offered (Stocco, Simonelli, Capra, & De Palo, 2012).

Guided by the Department of Psychology of the University of Padua in collaboration with two therapeutic communities in Venice (*Villa Emma* and *Villa Renata*) and run by a social cooperative (*Villa Renata*), the project “Research and intervention on minors in communities for drug-addicted mothers and their children: from at-risk parenting to child wellbeing” is an optimal example of how a multi-focused approach is necessary, in order to more widely evaluate psychological, social, and relational patterns in these women's lifestyles, involving, first of all, the net of family relationships that they are surrounded by, especially if children's wellbeing is also at risk (Stocco et al., 2012). Establishing and developing integrated approaches, focused on both intimate partner violence and substance abuse should therefore focus on interrupting the cycle of victimization, attempting to face all variables associated with them, at each level (Cohen et al., 2013).

An aspect that should be taken in consideration regards all those limitations of the psychiatric rehabilitation programs offered by psychiatric institutions that do not consider the gender issue. Given that the prevalence of co-occurring psychiatric disorders among substances abuse addicts has been identified to vary by genders, it appears relevant to orient interventions in order to address psychological and social needs more prevalent in women (Greenfield et al., 2007). Results from various studies (Compton, Cottler, Jacobs, Ben-Adballah, & Spitznagel, 2003; Mann, Hintz, & Jung, 2004; Westermeyer, Kopka, & Nugent, 1997) have been demonstrated on how the relationship between co-occurring psychiatric disorders, substance abuse treatment outcomes, and gender is highly complicated, and it might depend upon the sub-population involved, the features of the addiction, and the psychiatric disease considered (Greenfield et al., 2007). The simultaneous presence of different patterns would therefore make it more difficult to delineate psychiatric programs and treatment facilities specifically addressed and organized due to a gender perspective (Greenfield et al., 2007). Considering the range of variables which have a role in influencing treatment retention and completion, it is easy to understand why the comparisons of women-only versus mixed-gender treatment contexts is still so difficult to interpret (Greenfield et al., 2007).

## Conclusions

The latest researchers have underlined the interplay between intimate partner violence and substance abuse, a bidirectional relationship in which both patterns “create a cycle of spiraling losses and increasing vulnerabilities” (El-Bassel et al., 2005). Particularly, it is raising a growing recognition over the mental, emotional, and physical impact these factors have on women's health, whilst also considering all those consequences they have in the social and economic network.

In light of the spread of these phenomena and, above all, given their role on embracing various aspects of women's life, a multidisciplinary and multi-agency approach should be planned, in order to provide interventions directed to each level involved, from the individual to the societal, through the relationship and community setting (Brentari et al., 2011).
